# Dosimetric benefits of placing dose constraints on the brachial plexus in patients with nasopharyngeal carcinoma receiving intensity-modulated radiation therapy: a comparative study

**DOI:** 10.1093/jrr/rru072

**Published:** 2014-08-30

**Authors:** Hailan Jiang, Heming Lu, Hong Yuan, Huixian Huang, Yinglin Wei, Yanxian Zhang, Xu Liu

**Affiliations:** 1 Department of Radiation Oncology, People's Hospital of Guangxi Zhuang Autonomous Region, 6 Taoyuan Road, Nanning City, 530021, Guangxi, P. R. China; 2 Department of Otorhinolaryngology, People's Hospital of Guangxi Zhuang Autonomous Region, 6 Taoyuan Road, Nanning City, 530021, Guangxi, P. R. China; 3 Department of Oncology, Liuzhou Worker's Hospital, Liuzhou 545005, P. R. China; 4 Department of Clinical Medicine, Guangxi Medical University, Nanning 530021, P. R. China

**Keywords:** brachial plexus, dose constraints, radiation injury, nasopharyngeal carcinoma, intensity-modulated radiation therapy, comparative study

## Abstract

This study aimed to evaluate whether placing dose constraints on the brachial plexus (BP) could provide dosimetric benefits in patients with nasopharyngeal carcinoma (NPC) undergoing intensity-modulated radiation therapy (IMRT). Planning CT images for 30 patients with NPC treated with definitive IMRT were retrospectively reviewed. Target volumes, the BP and other critical structures were delineated; two separate IMRT plans were designed for each patient: one set no restrictions for the BP; the other considered the BP as a critical structure for which a maximum dose limit of ≤66 Gy was set. No significant differences between the two plans were observed in the conformity index, homogeneity index, maximum dose to the planning target volumes (PTVs), minimum dose to the PTVs, percentages of the volume of the PTVnx and PTVnd receiving more than 110% of the prescribed dose, or percentages of the volume of the PTVs receiving 95% and > 93% of the prescribed dose. Dose constraints significantly reduced the maximum dose, mean dose, V45, V50, V54, V60, V66 and V70 to the BP. Dose constraints significantly reduced the maximum dose to the BP, V45, V60 and V66 in both N0–1 and N2–3 disease; however, the magnitude of the dosimetric gain for each parameter between N0–1 and N2–3 disease was not significantly different, except for the V60 and V66. In conclusion, placing dose constraints on the BP can significantly decrease the irradiated volume and dose, without compromising adequate dose delivery to the target volume.

## INTRODUCTION

Intensity-modulated radiation therapy (IMRT) has been widely applied in the field of radiation oncology over the last decade and is considered the standard primary approach for nasopharyngeal carcinoma (NPC) [1]. The main advantages of IMRT are improved local/regional control through delivering a higher dose to the tumor targets, while decreasing the incidence or severity of radiation-induced injury by minimizing unnecessary irradiation of the surrounding normal tissues. Numerous studies have concluded that, compared with conventional RT, IMRT is capable of achieving a better target coverage and reducing the radiation dose to the central nervous system and other critical structures in patients with head-and-neck cancer (HNC) [2–5]. For patients with NPC, critical structures (including the parotid glands, spinal cord, brain stem, pituitary gland, temporal lobes, cranial nerves, and middle and inner ears) are inevitably exposed to unnecessary irradiation due to their close proximity to the primary tumor and/or metastatic lymph nodes. Therefore, in order to avoid excessive radiation doses, these organs at risk (OARs) are often considered as dose-limiting structures when designing IMRT treatment plans. The brachial plexus (BP) has been deemed as particularly important in these abovementioned structures. Radiation-induced brachial plexopathy (RIBP) has been reported in the treatment of breast cancer, lung cancer, lymphoma and other malignances involving the neck, shoulder or upper thorax [6–10]. The pathogenesis of RIBP may be direct radiation injury of the nerve or entrapment of the nerve fibers as a result of fibrosis of the surrounding connective tissues [9]. RIBP is believed to be a progressive process that may eventually result in devastating functional consequences, and no effective modality is yet available to treat this disease. Therefore, methods of preventing BP injury before its onset would appear to be particularly crucial. However, little attention has been paid to the BP in patients with NPC receiving IMRT, and data on dosimetric analysis of the BP in the literature is rare. In this study, we retrospectively analyzed the differences in the dosimetric parameters of the BP when it was and was not considered a dose-limiting structure, to evaluate whether placing dose constraints on the BP could provide a dosimetric benefit.

## MATERIALS AND METHODS

### Patient characteristics

From September 2011 to December 2012, 40 patients with newly diagnosed NPC underwent IMRT using an Elekta Synergy linear accelerator (Elekta, Stockholm, Sweden) at the Department of Radiation Oncology at the People's Hospital of Guangxi Zhuang Autonomous Region. Of these, seven patients treated with conventional radiation therapy followed by IMRT and three patients treated with palliative intent were excluded from the present study. The remaining 30 patients, who were treated with a definitive step-and-shoot IMRT technique, were included in this analysis. Of these patients, 25 were male and 5 were female, with a median age of 44 years (range, 22–67 years). All patients were pathologically diagnosed as having undifferentiated non-keratinizing carcinoma, and were staged according to the 2009 AJCC Staging System (Table 1). Written informed consent was obtained from all patients. This study was approved by the Institutional Review Board (IRB) of the People's Hospital of Guangxi Zhuang Autonomous Region.

**Table 1.** Patient characteristics

Characteristic	Value
Age, years	
Median	44
Range	22–67
Gender, *n* (%)	
Male	25 (83.3)
Female	5 (16.7)
T stage, *n* (%)	
T1	3 (10.0)
T2	13 (43.4)
T3	10 (333.3)
T4	4 (13.3)
N stage, *n* (%)	
N0	7 (23.3)
N1	4 (13.3)
N2	13 (43.4)
N3	6 (20.0)
AJCC stage group, *n* (%)	
I	1 (3.3)
II	6 (20.0)
III	15 (50.0)
IV	8 (26.7)
Radiation dose, Gy	
PTVnx	68.2
PTVnd	68.2
PTV1	62.0
PTV2	55.8
Chemotherapy, *n* (%)	
None	4 (13.3)
Induction + concurrent	14 (46.7)
Concurrent	12 (40.0)

### Delineation of targets and critical structures

Planning CT images for the 30 patients were retrieved from our medical database and transferred to Pinnacle treatment-planning software (Pinnacle; Philips Radiation Oncology Systems, Fitchburg, WI, USA). The target delineation protocols for NPC have been reported previously [11]. Briefly, the primary gross tumor volume (GTVnx) and the involved lymph nodes (GTVnd) were included as gross disease. Margins of 5–10 mm and 5 mm were added to the GTVnx and GTVnd, respectively, to form the corresponding clinical target volumes (CTVnx and CTVnd); the CTV1 was defined as the entire nasopharynx, parapharyngeal space, pterygopalatine fossa, posterior third of the nasal cavity and maxillary sinuses, inferior sphenoid sinus, posterior ethmoid sinus, skull base and anterior half of the clivus. The CTV1 also included the bilateral retropharyngeal lymph nodes and ipsilateral level II for the node-negative neck. The CTV1 extended to the next ipsilateral level for the node-positive neck, or included the full length of the ipsilateral neck for the node-positive lower neck. The CTV2 was defined as the low-risk node region below the CTV1. Level V was defined as the border between the CTV1 and CTV2. Level Ib was not included unless ipsilateral level IIA was involved. The respective planning target volumes (PTVs) were generated with a 5-mm margin.

**Fig. 1.**
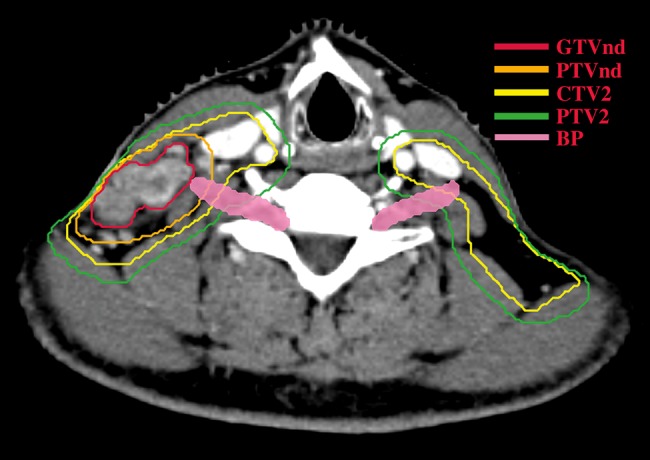
Example of the contoured targets and BP in a patient with T3N2M0 disease.

The contoured critical structures included the brain stem, optic chiasm, optic nerves, spinal cord, eyes, lens, cochlea, parotid glands, oral cavity, larynx, mandible, temporomandibular joints, BP and other critical normal tissues adjacent to the target volumes. For the BP, step-by-step delineation was performed following the CT-based atlas for delineation of the BP; these guidelines were proposed by Hall *et al*. [12] and endorsed by the Radiation Therapy Oncology Group (RTOG). An example of the contoured targets and BP in a patient with T3N2M0 disease is presented in Fig. 1.

### Dose prescription and dose constraints to the BP

Two separate IMRT plans were designed for each patient using the Pinnacle treatment planning software: Plan 1 did not use any restrictions for the BP, and Plan 2 considered the BP as a critical structure to which dose constraints were applied. The maximum dose limit to the BP was ≤66 Gy. The dose constraints for other critical structures were within the tolerance limits, according to the RTOG 0225 protocol. The doses delivered to the PTVnx, PTVnd, PTV1 and PTV2 were 68.2 Gy, 68.2 Gy, 62.0 Gy and 55.8 Gy, respectively, over 31 fractions (Table 1). The IMRT plans were normalized so that more than 95% of the PTVs received the prescribed dose, with the goal of no more than 1% of the PTVs receiving less than 93% of the prescribed dose, and no more than 10% of the PTVnx and PTVnd receiving more than 110% of the prescribed dose. A higher priority was given to the targets if target coverage and dose constraints for the BP could not be achieved simultaneously.

### Statistical analysis

Paired-sample *t*-tests (for normally distributed data) or the Wilcoxon signed-rank test (for data with a skewed distribution) were used to examine the differences in each parameter (i.e. the minimum, mean and maximum doses to the BP and irradiated volumes of the BP at different dose levels in the two plans). Mean ± standard deviation values are presented for normally distributed data and median (interquartile range) values for data with a skewed distribution. All statistical tests were two-sided; *P* < 0.05 was considered statistically significant. Analyses were performed using Microsoft Office Excel (Version 2007; Microsoft, Redmond, WA, USA) and SPSS software (SPSS 17.0, SPSS, Inc., Chicago, IL, USA).

## RESULTS

### Dosimetric parameters of the target volumes

No significant differences were observed in the conformity index (CI), homogeneity index (HI), maximum dose to the PTVs, minimum dose to the PTVs, percentage volumes of the PTVnx and PTVnd receiving more than 110% of the prescribed dose, and percentage volumes of the PTVs receiving 95% and > 93% of the prescribed dose between the two plans (Table 2).

**Table 2.** Differences in the dosimetric parameters of the target volumes between the two plans

Parameter	Plan 1	Plan 2	*P*-value
PTVnx			
CI	1.0004 (1.0000–1.0017)	1.0004 (1.0000–1.0019)	0.601
HI	1.123 ± 0.031	1.122 ± 0.030	0.794
Dmax (Gy)	76.557 ± 2.111	76.512 ± 2.015	0.794
Dmin (Gy)	60.537 ± 7.396	61.006 ± 7.010	0.055
V75.02 (%)	0.175 (0.000–2.690)	0.140 (0.000–4.490)	0.745
V64.79 (%)	99.705 ± 0.631	99.628 ± 0.866	0.167
V63.42 (%)	100.000 (99.930–100.000)	100 .000 (99.960–100.000)	0.864
PTVnd			
CI	1.004 ± 0.008	1.007 ± 0.012	0.114
HI	1.115 (1.098–1.137)	1.124(1.106–1.142)	0.265
Dmax (Gy)	76.065 (74.870–77.530)	76.630 (75.400–77.890)	0.265
Dmin (Gy)	59.075 (52.380–63.770)	58.565 (51.020–63.050)	0.565
V75.02 (%)	0.270 (0.000–0.750)	0.280 (0.010–2.680)	0.386
V64.79 (%)	99.563 ± 0.790	99.305 ± 1.1485	0.109
V63.42 (%)	99.960 (99.760–100.000)	99.935 (99.690–100.000)	0.365
PTV1			
CI	0.980 (0.010–2.680)	1.007 (1.000–1.013)	0.117
HI	1.230 (1.213–1.272)	1.237 (1.218–1.273)	0.265
Dmax (Gy)	76.230 (75.200–78.890)	76.720 (75.510–78.940)	0.265
Dmin (Gy)	46.595 (32.820–57.760)	46.690 (34.350–58.160)	0.086
V58.9 (%)	99.160 (98.780–99.990)	99.325 (98.770–99.990)	0.100
V57.66 (%)	99.510 (99.170–100.000)	99.590 (99.20–100.000)	0.485
PTV2			
CI	1.005 ± 0.005	1.007 ± 0.007	0.075
HI	1.267 ± 0.102	1.274 ± 0.104	0.094
Dmax (Gy)	70.694 ± 5.678	71.068 ± 5.800	0.094
Dmin (Gy)	44.325 (33.230–49.460)	41.720 (31.850–49.060)	0.424
V53.01 (%)	99.507 ± 0.523	99.342 ± 0.647	0.073
V51.89 (%)	99.840 (99.460–99.990)	99.730 (99.410–99.990)	0.096

PTVnx = planning target volume for primary disease, CI = conformity index, HI = homogeneity index, Dmax = maximum dose, Dmin = minimum dose, Vx = percentage of the volume of the target receiving a dose greater than *x* Gy, PTVnd = planning target volume for nodal disease, PTV1 = planning target volume for clinical target volume 1, PTV2 = planning target volume for clinical target volume 2.

Mean ± standard deviation is presented for normally distributed data; median (interquartile range) for data with a skewed distribution.

### Dosimetric parameters of the BP

There were significant differences in the maximum dose and mean dose to the BP between the two plans (70.20 ± 4.34 Gy vs 67.07 ± 4.33 Gy, *P* < 0.0001; 51.79 ± 5.30 Gy vs 49.98 ± 5.87 Gy, *P* < 0.0001). However, no significant differences were observed in the minimum dose to the BP between the two plans. In Plan 1, the maximum dose ranged from 61.05–77.71 Gy, and the proportions of patients in which the BP received a dose greater than 60 Gy, 66 Gy, and 70 Gy were 100% (30/30), 83.3% (25/30) and 46.7% (14/30), respectively; whereas in Plan 2, the maximum dose ranged from 58.72–74.98 Gy, and the proportions of patients in which the BP received a dose greater than 60 Gy, 66 Gy and 70 Gy were 93.9% (28/30), 60.0% (18/30) and 33.3% (10/30), respectively.

### Volumes of the BP irradiated at different dose levels

**Fig. 2.**
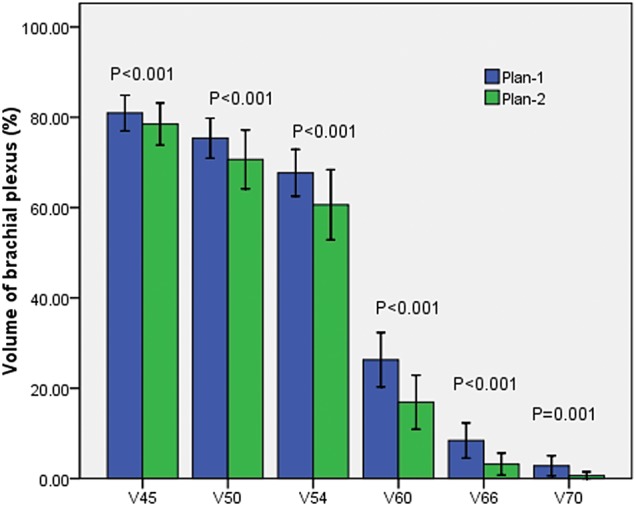
Irradiated volumes of the brachial plexus at different dose levels.

The V45, V50, V54, V60, V66 and V70 values are the percentage of the volume of the BP that received a radiation dose exceeding 45, 50, 54, 60, 66 and 70 Gy, respectively. Significant differences were observed in the V45, V50, V54, V60, V66 and V70 values between the two plans [81.575% (74.272–88.650%) vs 80.020% (68.537–88.125%), *P* < 0.001; 77.015% (67.967–84.497%) vs 72.880% (60.305–83.685%), *P* < 0.001; 68.685% (59.082–77.450%) vs 62.360% (46.245–75.810%), *P* < 0.001; 26.300 ± 16.101% vs 16.911 ± 15.995%, *P* < 0.001; 4.740% (0.412–11.555%) vs 0.275% (0.000–2.772%), *P* < 0.001; 0.070% (0.000–2.485) vs 0.000 (0.000–0.032), *P* = 0.001] (Fig. 2).

### Dosimetric analysis of the BP in N0–1 disease and N2–3 disease

When the BP was considered as a dose-limiting structure during treatment planning, significant reductions in both the dose of radiation and the irradiated volume of the BP were observed in patients with N0–1 disease and patients with N2–3 disease, with respect to several dosimetric parameters including the maximum dose, V45, V50, V54, V60 and V66. However, the magnitude of the dosimetric gain obtained in each parameter by applying dose constraints to the BP was not significantly different between N0–1 disease and N2–3 disease, except for the V60 and V66 (Table 3 and Table 4).

**Table 3.** Dosimetric analysis for the brachial plexus according to N stage

Parameter		Plan 1	Plan 2	*P*-value
N0–1	Dmax (Gy)	67.974 ± 4.169	65.002 ± 3.838	< 0.001
	V45(%)	82.550 (79.030–88.800)	80.160 (76.990–88.710)	0.002
	V50(%)	78.210 (73.000–85.050)	76.70 (66.940–85.350)	0.003
	V54(%)	72.466 ± 10.389	66.709 ± 14.317	0.004
	V60(%)	21.589 ± 13.862	11.146 ± 14.391	< 0.001
	V66(%)	1.080 (0.000–4.990)	0.000 (0.000–0.010)	0.012
			
N2–3	Dmax (Gy)	71.750(69.700–74.070)	69.510(65.495–71.065)	< 0.001
	V45(%)	78.350 (69.050–88.600)	76.150 (63.910–87.7200)	0.003
	V50(%)	74.780 (61.160–82.800)	70.940 (54.010–83.300)	0.003
	V54(%)	67.090 (48.350–75.660)	60.950 (42.960–73.120)	0.004
	V60(%)	29.028 ± 17.016	20.249 ± 16.281	0.001
	V66(%)	8.110 (2.310–12.590)	1.440 (0.000–4.010)	< 0.001

**Table 4.** Dosimetric gain for each parameter obtained by applying dose constraints to the brachial plexus in patients with N0–1 disease and patients with N2–3 disease

Parameter	Dosimetric gain^a^		*P*-value
	N0–1	N2–3	
Dmax (%)	4.327 ± 2.524	4.478 ± 3.620	0.904
V45 (%)	0.408 (0.206–3.371)	1.009 (0.018–4.104)	0.866
V50 (%)	1.130 (0.873–7.132)	2.342 (0.115–11.514)	0.703
V54 (%)	8.556 (2.144–12.301)	5.397 (0.573–22.669)	0.933
V60 (%)	63.112 ± 31.579	36.825 ± 34.198	0.046
V66 (%)	98.333 (90.692–100.000)	78.488 (48.258–92.016)	0.049

^a^Dosimetric gain for each parameter was calculated as follows: dosimetric gain = [(V1 – V2) ÷V1] × 100%, where V1 is the value in Plan 1, and V2 is the corresponding value in Plan 2.

## DISCUSSION

In our previous retrospective study [13], we separately delineated the right and left BP and recalculated the dose distributions, and found that, in a large proportion of patients, the BP was exposed to a higher dose when it was not outlined. The maximum dose to the left BP was 59.12–78.47 Gy, and the V60, V66 and V70 were 96.4%, 57.1% and 25.0%, respectively; and the maximum dose to the right BP was 59.74–80.31 Gy, and the V60, V66 and V70 were 96.4%, 64.3% and 39.3%, respectively. These results are consistent with our present study, in which the maximum dose to the BP ranged from 61.05–77.71 Gy, and the proportions of patients in which the BP received a dose greater than 60 Gy, 66 Gy and 70 Gy if no dose constraints were applied were 100% (30/30), 83.3% (25/30) and 46.7% (14/30), respectively. McGary *et al*. [14] evaluated patients with HNC, and reported that the doses to the BP were close to the target dose (66–70 Gy) when this OAR was not defined as a dose-limiting structure. Similar findings were obtained in a retrospective study conducted by Millender *et al*. [15], in which a total of 16 patients with HNC were treated with definitive IMRT, and the maximum dose to the BP ranged from 61.7–78.5 Gy, with a median dose of 68.5 Gy and a mean dose of 69.6 Gy.

The high radiation dose received by the BP in patients with HNC could be due to a number of factors. First, anatomically, a large proportion of the BP is located within the neck area. The BP originates from the spinal nerves exiting the spinal canal through the neural foramina from the C4–5 interspace to the T1–2 interspace, travels inferiorly between the anterior and middle scalene muscles and then traverses the interscalene triangle and costoclavicular space. The BP continues to extend within the space between the pectoralis minor muscle and anterosuperior chest wall, where it finally surrounds the axillary artery laterally to the pectoralis minor muscle [14]. Secondly, in patients with HNC, the bilateral neck is usually irradiated prophylactically or therapeutically. Thus the BP may be exposed to a large radiation dose due to its close proximity to the targets. Finally, in patients with metastasis to the lower neck, a higher tumoricidal dose needs to be delivered to the involved lymph nodes and the surrounding areas to achieve optimal tumor control. In this situation, the dose to the BP could be close to or even higher than the target dose. Millender *et al*. (15) found that the median maximum-point dose in patients with HNC was higher when grossly positive nodes were adjacent to the BP (71.9 Gy) than when it was adjacent to node-negative regions (65.7 Gy). This phenomenon is more evident in patients with NPC. As a unique subtype of HNC, NPC has a higher risk of metastasis to the neck lymph nodes, with an incidence of 85–90% in the ipsilateral neck and 50% in the bilateral neck. For this reason, the whole neck is usually covered by the clinical target volume, and the tumoricidal radiation dose needs to be delivered to the positive lymph nodes. Delivery of radical radiation to gross nodal disease may increase the risk of the BP receiving an excessive dose [16]. In a previous study, we found that the maximum dose to the BP adjacent to positive lymph nodes (BPAN) was significantly higher than to a BP not adjacent to positive lymph nodes (BPNAN; 72.84 ± 3.91 Gy vs 64.81 ± 3.47 Gy on the left side, *P* < 0.001; 72.91 ± 4.74 Gy vs 64.91 ± 3.52 Gy on the right side, *P* < 0.001), reflecting the close proximity of the nodal regions to the BP. In addition, for the bilateral BP, BPANs had significantly higher V60 and V66 values compared with BPNANs. In the present study, the maximum dose to the BP without applying dose constraints in N2–3 disease was 71.7500 Gy (69.7000–74.0700 Gy), suggesting a higher radiation dose would be delivered to the BP in patients with advanced N stage disease. In fact, a higher maximum dose was delivered to the BP when no dose constraints were used, even in patients with early N stage (N0–1) disease.

Several researchers have evaluated dosimetric outcomes by applying dose constraints to the BP during treatment planning. Truong *et al*. [17] retrospectively analyzed 114 patients with HNC that underwent IMRT receiving a total dose of 69.96 Gy over 33 fractions. During IMRT optimization, the intent was to keep the maximum dose to the BP to ≤60 Gy, yet prioritizing tumor coverage over achieving dose constraints for the BP. The study demonstrated that dose constraints for the BP were difficult to achieve at ≤60 to 66 Gy in T4 disease of the larynx, hypopharynx, and oropharynx or in N2/3 disease. In patients with HNC treated with definitive or postoperative IMRT, Chen *et al*. [18] found that the maximum dose to the ipsilateral BP ranged from 0–81 Gy (median, 63 Gy); the mean dose ranged from 0–69 Gy (median, 56 Gy), and the mean V60, V66, V70 and V74 values for the ipsilateral BP were 39%, 28%, 6% and 3%, respectively.

However, neither of the studies described above were comparative. Whether a treatment plan designed to spare the BP provides dosimetric benefits compared with a plan which does not specifically protect the BP was previously unknown. To the best of our knowledge, the present study was the first to compare the dosimetric parameters for the BP when it was/was not considered a dose-limiting structure in patients with NPC treated using IMRT. We found that application of dose constraints significantly decreased the maximum and mean doses to the BP. Similar reductions were also observed for other dosimetric parameters, including the V45, V50, V54, V60, V66 and V70. These advantages occurred in both patients with N0–1 disease and patients with N2–3 disease, indicating that the dosimetric benefits obtained placing dose constraints on the BP were not limited to either early or advanced N stage. In addition, sparing of the BP did not compromise adequate dose delivery to the target volumes. However, with regard to the magnitude of the dosimetric gain, patients with N0–1 disease obtained more benefit at a higher dose level (V60 and V66) than patients with N2–3 disease.

The risk factors for developing RIBP after radiotherapy include the total radiation dose, fraction size and radiation technique, according to analyses mainly based on patients with breast cancer [19–21]. In HNC, Chen *et al*. [18] found that both the maximum dose and V74 were independently predictive of brachial plexopathy. Another study of a large series at the same institution found that neck dissection and the maximum radiation dose were independent predictors of the symptoms of BP-associated neuropathies. In addition, a modeled relationship for the probability of developing neuropathic symptoms and the maximum radiation dose to the BP was established; each Gy increase in the maximum dose was associated with a 1.39-fold increase in the odds of developing symptoms. In particular, the rates of BP injury increased dramatically above doses >70 Gy [22, 23], suggesting a threshold effect. However, this does not mean that RIBP can be totally avoided when the maximum dose is ≤70 Gy. Asymptomatic changes may be more pronounced than clinical manifestations at a lower dose level, as demonstrated in a study by Boyacyian *et al*. [24], in which significant electrophysiological changes were observed after one year in the BP of patients who received a dose of 54 Gy. Based on these findings, the optimal strategy for preventing RIBP in patients with NPC should aim to keep the radiation dose to the BP as low as possible.

## CONCLUSION

In conclusion, placing dose constraints on the BP in patients with NPC during IMRT treatment planning could significantly decrease the irradiated volumes and radiation dose for this OAR, without compromising adequate dose delivery to the target volumes.

## FUNDING

The present study was financed by grants from the Guangxi Sci–Tech Office (No. 0816004-40) and the Guangxi Health Department (Z2013370), China. Funding to pay the Open Access publication charges for this article was provided by the People's Hospital of Guangxi Zhuang Autonomous Region, China.
